# Does Treatment with Sodium-Glucose Cotransporter-2 Inhibitors Affect Adherence to International Society Criteria for Diabetic Ketoacidosis in Adult Patients with Type 2 Diabetes? A Retrospective Cohort Analysis

**DOI:** 10.1155/2024/1849522

**Published:** 2024-03-14

**Authors:** Aongus O'Brolchain, Joshua Maletsky, Ibrahim Mian, Serena Edwards

**Affiliations:** ^1^Department of Medicine, Gold Coast Health, Gold Coast, Queensland, Australia; ^2^Griffith University, Queensland, Australia

## Abstract

**Design:**

Retrospective observational study. *Setting*. Inpatients at two teaching hospitals in Queensland, Australia. *Primary Outcome Measure(s)*. The number of patients meeting the Joint British Diabetes Society (JBDS) and American Association of Clinical Endocrinology/American College of Endocrinology (AACE/ACE) diagnostic criteria for DKA. Patients were divided into two groups by treatment with SGLT2i at the time of diagnosis. *Participants*. Adult patients (>18 years old) with type 2 diabetes diagnosed with DKA from April 2015 to January 2022. Patients without type 2 diabetes were excluded.

**Results:**

One hundred and sixty-five patients were included in this study—comprising 94 patients in the SGLT2i cohort and 70 in the non-SGLT2i cohort. A significantly smaller proportion of patients in the SGLT2i vs. non-SGLT2i cohorts met both JBDS (56% vs. 72%, *p* = 0.035) and AACE/ACE (63% vs. 82%, *p* = 0.009) criteria for diagnosis of DKA.

**Conclusion:**

Patients with type 2 diabetes treated with SGLT2i may be more likely to be diagnosed with DKA despite not meeting the criteria. Despite recent adjustments to account the physiological effects of SGLT2i, significant variation in criteria between major society guidelines presents ongoing challenges to clinicians. The proportion of patients diagnosed using both JDBS and AACE/ACE were comparable, suggesting a reasonable degree of agreement.

## 1. Introduction

Diabetic ketoacidosis (DKA), historically defined by the triad of hyperglycaemia, ketosis, and metabolic acidosis, is a potentially life-threatening complication of diabetes mellitus that is caused by absolute or relative insulin deficiency. DKA predominantly affects patients with type 1 diabetes, and until recently, it was regarded as rare in patients with type 2 diabetes [1]. Sodium-glucose transporter inhibitors (SGLT2i) are an established oral treatment in the management of (predominantly) type 2 diabetes. SGLT2i induce glucosuria, lowering serum glucose levels and therefore insulin secretion, resulting in relative insulin deficiency [1, 2]. In 2015, the Food and Drug Administration issued a warning relating to a possible increase in the risk of “euglycaemic” DKA (EuDKA) associated with the use of SGLT2i. Meta-analyses have confirmed this rare association [3].

True euglycaemia (blood glucose < 7.8 mmol/L) in patients presenting with SGLT2i-related DKA is relatively rare, prompting the American Association of Clinical Endocrinology/American College of Endocrinology (AACE/ACE) to recommend against the use of the term “euglycaemic ketoacidosis,” instead favouring the term “DKA with lower-than-anticipated glucose levels” [4].

Until recently, simply establishing the presence of ketosis by demonstrating ketonuria (acetoacetate) using the semiquantitative “nitroprusside reaction” was sufficient to satisfy the criteria for the diagnosis of DKA [5]. This method is no longer recommended, particularly in the presence of SGLT2i, because these medications lead to reabsorption of ketone bodies in the renal tubules [6]. Beta-hydroxybutyrate (B-OHB) and acetoacetate are the predominant ketones involved in ketoacidosis, with circulating levels of B-OHB reaching ten times that of acetoacetate once DKA is established. Moreover, during the resolution of ketoacidosis following treatment, B-OHB is reduced to acetoacetate and excreted in the urine. Thus, there is a paradoxical increase in ketonuria (specifically acetoacetate), giving the erroneous impression of worsening ketoacidosis and failure of treatment [7].

Hyperglycaemia and ketonuria are thus no longer reliable tools for the diagnosis of DKA in patients taking SGLT2i, creating diagnostic challenges for clinicians. Adding to the uncertainty, there is heterogeneity in published guidelines from the major endocrinology societies regarding diagnostic criteria and cut-offs for DKA.

**Figure 1:**
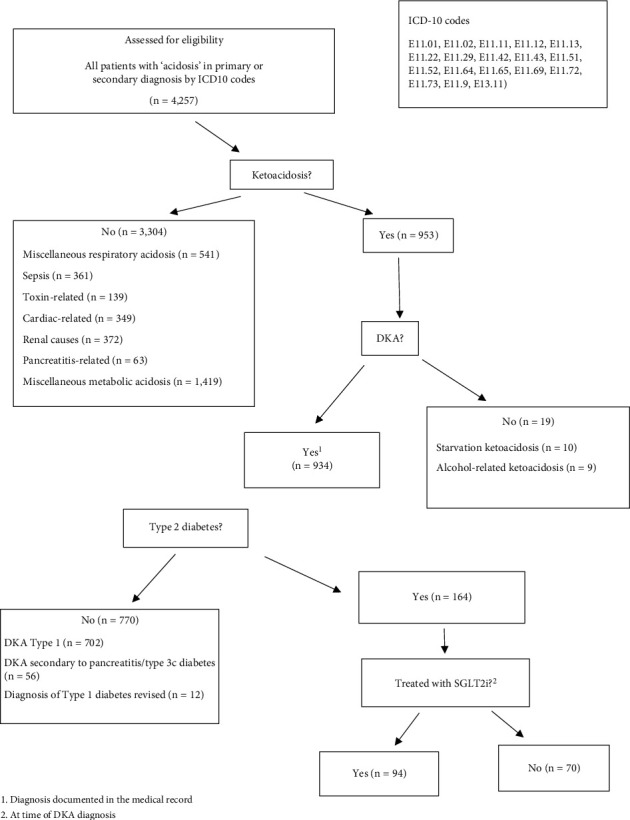
Illustration of how patients were selected for inclusion following initial screening using ICD-10 codes. All cases of nondiabetic ketoacidosis were excluded. ^1^Diagnosis documented in the medical record. ^2^At the time of DKA diagnosis.

Some of the major endocrine and diabetes society criteria (see Figure 1) have issued updates to their criteria to account for SGLT2i. Since 2011, the Joint British Diabetes Society (JBDS) guidelines have advocated for a blood glucose level (BGL) threshold of >11 mmol/L or a history of diabetes [6, 8]. The American Association of Clinical Endocrinology/American College of Endocrinology (AACE/ACE) suggest hyperglycaemia (13.9 mmol/L) or lower glycaemia in specific cases (cut-offs not specified) [4]. The American Diabetes Association (ADA) guidelines for diagnosis and management of DKA have not been updated since 2009, and although serum (and urine) ketone testing is recommended, a threshold is not specified [9]. Important differences between guidelines are the absence of bicarbonate as a diagnostic criterion in the AACE/ACE guidelines and omission of anion gap (AG) in the JBDS guidelines [6]. Other important discriminators between guidelines are the inclusion of “history of diabetes” (JBDS) and “lower glycaemia in specific cases” (AACE/ACE) rather than a fixed threshold for hyperglycaemia [4, 6]. Despite these differences, adherence to diagnostic criteria (i.e., the demonstration of a metabolic acidosis with commensurate levels of blood ketones) is increasingly important given both the obsolescence of previously used methods in the diagnosis of DKA and the lower-than-expected glycaemia induced by SGLT2i.

DKA caused by SGLT2i is very rare, with one meta-analysis of clinical trials finding no increased risk for DKA versus placebo [10]. However, a considerable number of publications relating to the association of SGLT2i and DKA exist in the literature [11]. This study examined the biochemical parameters used to diagnose DKA in patients with type 2 diabetes and investigated whether they met international society criteria (JBDS, ACE/AACE) and whether treatment with SGLT2i may have affected the threshold for diagnosis.

## 2. Methods

### 2.1. Participants

This study was conducted at the Gold Coast University and Robina Hospitals in Queensland, Australia. All patients with a diagnosis of DKA from 1st April 2015 to January 2022 were screened using the International Statistical Classification of Diseases and Related Health Problems, 10th Revision (ICD-10) codes from the electronic medical record (ethics approval: LNR/2022/QGC/86036). These included patients presenting to the emergency department, those referred from outpatient procedures, and inpatients. To maximize the number of patients captured, charts were screened for acidosis (E87.2), type 2 diabetes with ketoacidosis (E11.11, E11.01, E11.02, E11.73, and E11.65,) type 2 diabetes with hyperosmolarity (E10.0), other specified diabetes mellitus with ketoacidosis without coma (E13.10, E.13.11), disorders of ketone metabolism (E71.32), and ketosis (E88.8). The remaining ICD-10 codes are shown in Figure 1.

### 2.2. Eligibility Criteria

All patients 18 years or older with an established or new diagnosis of type 2 diabetes diagnosed with DKA were included. Charts were reviewed from the electronic medical record by four investigators (AOB, JM, IM, and SE). Patients with respiratory and miscellaneous causes of nondiabetic metabolic acidosis were excluded. Patients with an established diagnosis of type 1 diabetes and type 3c diabetes, those under 18 years old, and patients with ketoacidosis not related to diabetes were excluded. Index presentations of type 1 diabetes and patients with a revised diagnosis (from type 2 to type 1) were excluded. Following application of the eligibility criteria, medication lists at the time of admission were reviewed. Patients were then divided into two groups: those taking SGLT2i and those not taking SGLT2i at the time of diagnosis (Figure 1).

### 2.3. Outcome Measures

**Figure 2:**
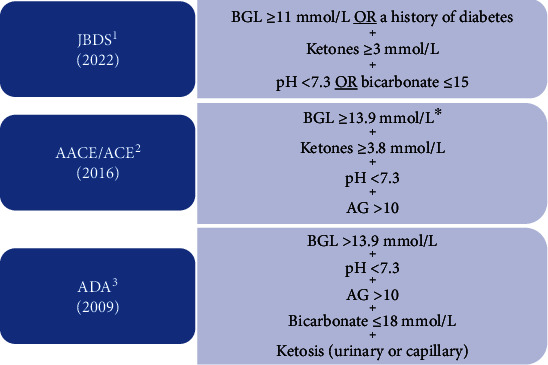
Adapted from ^1^Joint British Diabetes Societies [6], ^2^American Association of Clinical Endocrinologists/American College of Endocrinology [4], and ^3^American Diabetes Association [9]. ^∗^Lower in certain situations (threshold not specified). BGL = blood glucose level; AG = anion gap.

All information recorded was at the time of diagnosis of DKA. Confirmation of diagnosis of DKA was based on documentation in the medical record. Diagnoses of “true” DKA were based on published JBDS and ACE/AACE criteria (Figure 2).

#### 2.3.1. Diagnostic Criteria

Categorical diagnostic variables were created based on specific thresholds corresponding to the JBDS and ACE/AACE criteria: AG > 10 mol/L, bicarbonate < 15, pH < 7.3, ketones > 3.8 mmol/L, and ketones > 3 mmol/L.

To establish whether patients met JBDS criteria for diagnosis of DKA, a dichotomous variable was created, where the following criteria were required: ketones > 3 AND pH < 7.3 OR bicarbonate < 15. For ACE/AACE criteria, the following criteria were required: capillary ketones > 3.8 AND pH < 7.3 AND anion gap > 10. A threshold for hyperglycaemia was not included as a diagnostic criterion due to provisions made in the criteria to account for lower-than-expected glycaemia (Figure 2).

#### 2.3.2. Biochemical Parameters

Laboratory indices at time of presentation were recorded as continuous variables: lactate, anion gap, serum bicarbonate, HbA1c, eGFR at presentation, and baseline eGFR.

#### 2.3.3. Demographics and Medications

Baseline demographic information included age, sex, body mass index (BMI (kg/m^2^)) and weight (kg). BMI was also considered as a dichotomous variable (≤30 kg/m^2^, >30 kg/m^2^). Additional information gathered included medications on admission for DKA (insulin, metformin, SGLT2i, sulfonylureas, glucagon-like peptide-1 (GLP-1) agonists, and dipeptidyl peptidase 4 (DPP4) inhibitors), use of insulin infusion, admission to intensive care, history of DKA/hyperosmolar hyperglycaemic state (HHS), and length of hospital stay.

### 2.4. Data Analysis

Statistical analysis was performed using Stata 17 (College Station, TX, USA). Means and standard deviations were used to summarize continuous variables. For continuous variables with normal distribution, the *t*-test was used. For non-normally distributed continuous variables, the Mann–Whitney *U* test was used to compare groups by SGLT2i status. Frequencies and percentages were used for categorical variables. Fisher's exact test was used to test for association between SGLT2i status and categorical variables.

## 3. Results

A total of 4,257 patients were screened from the electronic medical record by retrospective chart review using ICD-10 codes (Figure 1). Following chart review by four investigators, 164 patients met the inclusion criteria. These patients were further subdivided into 2 groups: those with (*n* = 94) and without (*n* = 70) a documented history of current SGLT2i use at the time of DKA diagnosis.

### 3.1. Diagnostic Criteria

**Figure 3:**
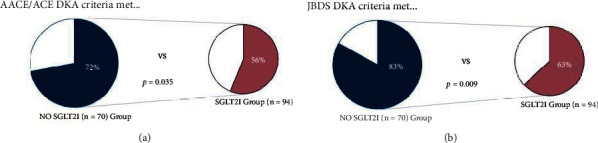
Proportions of patients in each group meeting AACE/ACE (a) and JBDS (b) criteria for DKA: SGLT2i group (red) on the right and the non-SGLT2i group (blue) on the left. AACE/ACE: American Association of Clinical Endocrinology/American College of Endocrinology; JBDS: Joint British Diabetes Society.

In both groups, a large proportion of patients did not meet the specified criteria for a diagnosis of DKA (Figure 3). A significantly smaller proportion of patients in the SGLT2i group met the criteria for DKA versus the non-SGLT2i group according to both the AACE/ACE (56% vs. 72%, *p* = 0.035) and JBDS guidelines (63% vs. 82%, *p* = 0.009). Using JBDS (<11 mmol/L) and AACE/ACE/ADA criteria (<13.9 mmol/L), “euglycaemia” was observed in 39% and 48%, respectively, of patients in the SGLT2i group vs. 3% in the non-SGLT2i group (*p* = <0.001).

### 3.2. Biochemical Parameters

Significant differences in pH, serum bicarbonate, and eGFR were observed between the SGLT2i and non-SGLT2i groups. Significant differences were observed in blood glucose levels ((median) 14.35 mmol/L [9, 12] vs. 26.25 mmol/L (21, 39), *p* = 5.3 × 10^−15^). The remainder of between-group differences is presented in Table 1.

### 3.3. Demographic Information

Between-group differences are presented in Table 2. No significant differences in age, sex, or duration of diabetes were observed between groups. Mean HbA1c levels were significantly lower in the SGLT2i group (9.3 (2.1) vs. 11.7 (2.7), *p* = 7.6 × 10^−7^). Antibodies (IA2, GAD, and ICA) were measured in 50/164 patients (30.4%).

### 3.4. Medications

Significant differences were observed between groups in the use of metformin (88.3% vs. 64.3%, *p* = 4.7 × 10^−4^), DPP4 inhibitors (34% vs. 12.9%, *p* = 0.002), and statins (68.1% vs. 38.6%, *p* = 2.4 × 10^−4^). Between-group differences are summarized in Table 1.

Figure 3 illustrates the proportion of patients in each group (the blue, left-sided pie charts representing those not treated with SGLT2i, and the red, right-sided pie-charts representing the SGLT2i-treated cohort) meeting both AACE/ACE criteria (a) and JBDS (b) criteria.

## 4. Discussion

This study demonstrated that adult patients with type 2 diabetes treated with SGLT2i may be more likely to be diagnosed with DKA despite not meeting the international diagnostic criteria compared to those not taking these medications at the time of diagnosis. It is not clear whether the known association between SGLT2i and DKA in type 2 diabetes affects the tendency of clinicians to adhere to international society guidelines. Differences observed between groups in the use of some oral antihyperglycaemic agents (metformin, DPP4i) and statins may reflect more index presentations of diabetes in the non-SGLT2i cohort. A significant difference in blood glucose levels between groups at presentation was expected, given the glucosuria induced by SGLT2i, and this has been observed in similar studies [13]. It is not clear whether the lower blood glucose levels resulted in fewer patients in the SGLT2i group being treated with an insulin infusion. Interestingly, there was no significant difference in AG between groups diagnosed with DKA, similar to previous studies [13]. Lower eGFR levels in the SGLT2i group may reflect the fact that fewer patients in this group met the criteria for DKA and were thus less unwell, given that there was no difference between groups in baseline eGFR.

Acidosis is the *sine qua non* of diabetic ketoacidosis, and the criterion of pH < 7.3 is common to the major society guidelines [4, 6, 9]. This criterion was present in just 79% of the non-SGLT2i group and 68% of SGLT2i cohort, suggesting a tendency toward over-/misdiagnosis of DKA in both groups. The differences observed in the severity of the acidaemia between groups may at least partly be explained by several patients being diagnosed in the periprocedural setting, with different criteria employed by the Australian and New Zealand College of Anaesthetists (ANZCA) to diagnose DKA. In 2022, the ANZCA, in consultation with the Australian Diabetes Society (ADS), published guidelines advising that a diagnosis of DKA should be made in patients where the base excess (BE) level (defined as the amount of acid or base required to restore a pH of 7.4 to a sample of whole blood *in vitro* under specific conditions [14]) is less than -5 mmol/L and a capillary ketone level of >1 mmol/L [10, 15]. SGLT2i are known to cause persistent but mild ketosis [16]. The capillary ketone threshold suggested by the ANZCA/ADS is significantly less than that recommended by the AACE/ACE and JBDS, and BE is not used as a guideline in any of the major endocrine society guidelines [6, 9, 17]. A recent study demonstrated that, in fasted patients without diabetes, capillary ketones of up to 1.7 mmol/L can be observed, causing the ADS to revise their periprocedural guidelines, with a previous reference range of <1 mmol/L being suggested [17–19]. It is therefore likely that procedures have been cancelled due to fasting ketosis which was in the physiological range. This study underscores the need for a more universal, interdisciplinary definition of DKA to avoid potential uncertainty and risk of unnecessary deferral of procedures.

In writing of the JBDS guidelines, one reason given for the omission of AG as a criterion by the authors is that aggressive fluid resuscitation with normal saline (NaCl 0.9%) can result in a hyperchloraemic AG metabolic acidosis that may erroneously be interpreted as a failure of treatment or nonresolution of ketoacidosis [6]. The threshold level of for ketonaemia in the JBDS, ACE/AACE, and the International Society for Paediatric and Adolescent Diabetes (ISPAD) is derived from an influential study that demonstrated that blood B-OHB of 3.0 and 3.8 mmol/L corresponded to a serum bicarbonate concentration of 18 mmol/L in children and adults, respectively [12, 20]. Attributing severe metabolic acidosis, for example, to the presence of mild ketonaemia (<2 mmol/L) in a patient treated with SGLT2i, may result in failure to exclude more likely causes and a misdiagnosis of DKA.

Indeed, it has been hypothesized that ketone bodies may have intrinsic antioxidative and anti-inflammatory properties and that low-grade ketosis may mediate the cardiorenal benefits demonstrated in large clinical trials [21]. More significant ketosis (B‐OHB > 3 mmol/L) without acidosis (pH > 7.3) may trigger unwarranted escalation in medical treatment. It is therefore of paramount importance to incorporate the diagnostic criteria into clinical decision-making.

DKA in patients with type 2 diabetes is rare [22], and SGLT2i-related DKA is even more so [23]. In a meta-analysis of 39 randomized-control trials, the absolute rate of ketoacidosis in patients with type 2 diabetes taking SGLT2i was shown to be 3 events per 1000 patient years [11]. This study indicates tendency to overdiagnose DKA in patients with type 2 diabetes irrespective of SGLT2i treatment. Despite the low incidence of SLGT2i-associated DKA, the presence of an SGLT2i in the medication chart may elicit the availability heuristic, reducing adherence to major society guidelines for diagnosis of DKA.

As the indications for SGLT2i expand beyond diabetes into chronic kidney disease and heart failure, rigorous adherence to existing diagnostic criteria for DKA will be necessary to avoid overdiagnosis with its attendant risks, including unnecessary deferral of procedures and adverse clinical outcomes associated with overtreatment (e.g., hypokalaemia, hypoglycaemia). Indeed, an erroneous label of “SGLT2i-related DKA” or “euglycaemic DKA secondary to SGLT2i” in a patient's medical history may result in reticence on the part of clinicians to continue or resume treatment with this important class of medications in the affected patients.

Strengths of this study include its relatively large sample size given the rareness of both DKA in type 2 diabetes and SGLT2i-related DKA, and cohort size was comparable to other similar studies [24, 25]. The study included all patients from 2015, when the initial FDA warning was published, which mitigated selection bias and maximized the number of cases for inclusion in the study. This study has limitations that must be considered. These include its retrospective and observational design. The use of ICD-10 codes to source patients is imperfect, and there is a risk of missing data, although this was partially mitigated by including for screening all patients with a diagnosis of “acidosis” and manual review of individual charts for a diagnosis of “diabetic ketoacidosis.” It was not possible to exclude all patients subsequently diagnosed with latent autoimmune diabetes of adulthood in this study. Additionally, patients were designated as having a history of SGLT2i use based on admission documentation alone, and it was not possible to corroborate this information in all cases. However, it is possible that the perception of SGLT2i use may influence clinician decision-making, resulting in increased vigilance and overdiagnosis of DKA.

## Figures and Tables

**Table 1:** Results of univariate analysis of biochemical parameters and medications prescribed (in the community) at the time of DKA diagnosis.

Variable	SGLT2i group	Non-SGLT2i group	*p* value
Biochemical parameters at diagnosis			
eGFR (mL/min)	57 (49, 68)	48 (31, 80)	0.03
pH	7.26 (7.2, 7.31)	7.215 (7.11, 7.28)	0.006
Anion gap	19.9 (6.7)	20.0 (6.9)	0.96
HCO3 (mEq/L)	15.0 (6.3)	13.1 (6.0)	0.05
Lactate (mmol/L)	1.9 (1.4, 2.6)	2.15 (1.5, 3.2)	0.20
HCO3 < 15 (mEq/L), *n* (%)	41 (43.6%)	52 (74.3%)	1.2 × 10^−4^
Anion gap > 10, *n* (%)	87 (93.6%)	67 (95.7%)	0.73
pH < 7.3, *n* (%)	64 (68.1%)	57 (81.4%)	0.072
Blood glucose (mmol/L) (mean)	14.35 (9, 20)	26.25 (21, 39)	5.3 × 10^−15^
Blood glucose > 11 mmol/L, *n* (%)	58 (61.7%)	68 (97.1%)	1.5 × 10^−8^
Blood glucose > 13.9 mmol/L, *n* (%)	50 (53.2%)	68 (97.1%)	3.2 × 10^−11^
Capillary ketones (mmol/L) (mean)	5.35 (3.2, 6.1)	5.6 (4.1, 6.5)	0.19
Ketones > 3.8 mmol/L, *n* (%)	66 (70.2%)	57 (82.6%)	0.097
Ketones > 3 mmol/L, *n* (%)	74 (78.7%)	61 (88.4%)	0.141
Medications at the time of diagnosis			
Metformin, *n* (%)	83 (88.3%)	45 (64.3%)	4.7 × 10^−4^
GLP-1 agonist, *n* (%)	8 (8.5%)	2 (2.9%)	0.19
Sulfonylurea, *n* (%)	15 (16.0%)	6 (8.6%)	0.24
Insulin, *n* (%)	43 (45.7%)	38 (54.3%)	0.34
Units (insulin)^1^	48 (35, 74)	43 (28, 64)	0.29
DPP4 inhibitor, *n* (%)	32 (34.0%)	9 (12.9%)	0.002
Statin, *n* (%)	64 (68.1%)	27 (38.6%)	2.4 × 10^−4^

Mean (SD), comparisons are by *t*-test. Median (Q1, Q3) where Q1 is the 1^st^ quartile and Q3 is the 3^rd^ quartile. Comparisons are by rank-sum test. ^1^For individuals being treated with insulin.

**Table 2:** Demographic information by SGLT2i treatment status.

Variable	SGLT2i (*n* = 94)	Non-SGLT2i (*n* = 70)	*p* value
Demographic information			
Sex, *n* (%)	F^∗^ 42 (44.7%)	F 37 (52.9%)	0.344
	M^∗∗^ 52 (55.32%)	M 33 (47.1%)	
Duration of diabetes (years)	13 (7, 20)	9 (3, 18.5)	0.057
Insulin infusion, *n* (%)	82 (87.2%)	68 (97.1%)	0.026
ICU admission, *n* (%)	17 (18.1%)	20 (28.6%)	0.13
BMI > 30, *n* (%)	27 (35.5%)	16 (31.4%)	0.70
Body mass index (BMI, kg/m^2^)	28.7 (5.8) (measured in 75/94 patients)	28.6 (7.2) (measured in 53/70 patients)	0.81
Previous DKA/HHS	10 (10.6%)	17 (24.6%)	0.020
Length of stay (days)	4 (2, 8)	5.5 (3, 10)	0.10
Age (years)	62.8 (12.6)	61.7 (17.1)	0.64
HbA1c (%)	9.3 (2.1)	11.7 (2.7)	7.6 × 10^−7^
Baseline eGFR (mL/min)	90 (78, 90)	90 (69, 90)	0.12

Mean (SD), comparisons are by *t*-test. Median (Q1, Q3) where Q1 is the 1^st^ quartile and Q3 is the 3^rd^ quartile. Comparisons are by rank-sum test. ^∗^Female. ^∗∗^Male.

## Data Availability

The data that support the findings of this study are available from the corresponding author upon reasonable request.
